# COVID-19 and Cybersecurity: Finally, an Opportunity to Disrupt?

**DOI:** 10.2196/21069

**Published:** 2021-05-06

**Authors:** Ana Ferreira, Ricardo Cruz-Correia

**Affiliations:** 1 CINTESIS Faculty of Medicine University of Porto Porto Portugal; 2 MEDCIDS Faculty of Medicine University of Porto Porto Portugal

**Keywords:** COVID-19, cybersecurity, challenges and disruption, data protection, privacy, health data

## Abstract

COVID-19 has challenged cybersecurity to meet the ultimate need of guaranteeing the privacy and security of human beings. Although personal and sensitive health data are needed to better understand, detect, and control the disease, many related cybersecurity challenges and vulnerabilities require further analysis and proper discussion. The aims of this viewpoint are to explore the consequences of COVID-19 on cybersecurity and health care as well as to foster awareness regarding the need for a change in paradigm on how cybersecurity is approached. Education and information technology literacy are important when they are suitably provided; however, they are certainly not a complete solution. Disruption needs to occur at the core of human-device interactions. Building trust, providing novel means to interact with technology (eg, digital humans), and supporting people—the most important cybersecurity asset—are only some of the recommendations for a more human and resilient approach to cybersecurity, during or after the pandemic.

## Introduction

The COVID-19 pandemic has created many difficult challenges and required many decisions to be made to quickly adapt to the situation on a daily basis. However, COVID-19 has serious consequences related to cybersecurity and the human right to privacy, security, and even physical integrity. Many of these consequences are directly related to the treatment of the disease, such as sharing of personal and sensitive data for research and treatment or contact tracing of patients; meanwhile, other consequences can be indirectly linked with the pandemic but are equally dangerous. These consequences include the continued treatment of other diseases while patients are confined to their homes and the increased vulnerabilities and risks of physical and web-based security when people rely on the internet for most of their daily activities (eg, working from home, homeschooling, shopping on the web, home banking, contact with friends and family, exercising, and entertaining).

Within the literature, it is recommended that data analyses should be performed in accordance with the law and with respect for privacy, which can increase public trust and adherence [1,2]. The need for transparency is even greater when personal and sensitive data are used for contact tracing using smartphone technology. Contact tracing apps are powerful tools that can help limit disease transmission during a pandemic, enforce quarantine rules, notify users of risk zones, or warn infected people [3]. However, contact tracing apps present significant privacy concerns because they collect personal data, such as location, which can also be used to perform a high degree of surveillance and harm individuals’ privacy [4]. An adequate balance between anonymity and data quality and integrity, with adequate transparency by certified authorities, is required.

Although many other diseases or conditions may require constant support and treatment, the COVID-19 pandemic has also exacerbated emergencies that may not be promptly addressed, such as chronic, oncological, or mental health conditions [1,5]. Health care professionals must opt for alternative (possibly less secure) means to support their patients, such as teleconsultation, email, and social networks [6].

These cybersecurity issues are just the beginning. In the first part of this viewpoint (COVID-19, Cybersecurity, Privacy, Security, and Safety), the authors identify and discuss cybersecurity challenges and consequences that COVID-19 has brought to the surface that need urgent attention. This part is based on our published work [7]. In the second part (Required Changes in Cybersecurity), recommendations for novel approaches to address the identified issues are advanced to foster a change in the current cybersecurity paradigm. This section comprises the authors’ original recommendations and are specific to this viewpoint.

## COVID-19, Cybersecurity, Privacy, Security, and Safety

**Figure 1:**
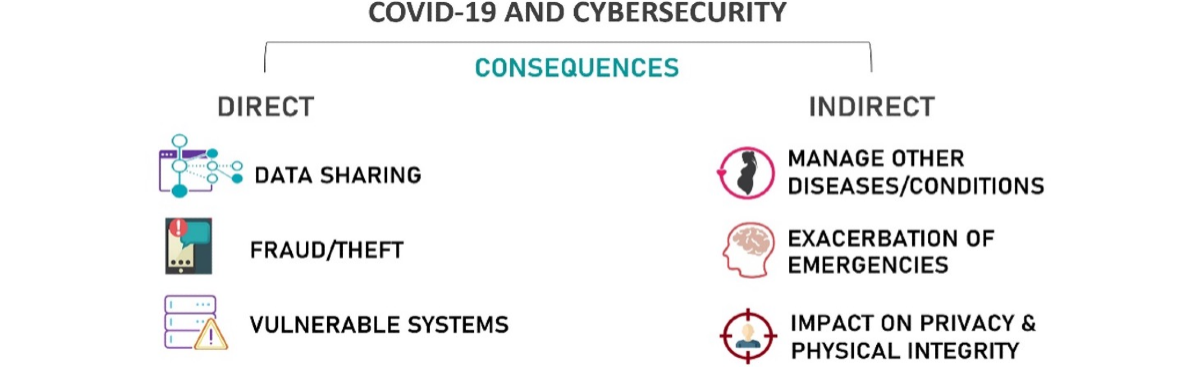
Direct and indirect consequences of COVID-19 for cybersecurity as identified by the authors.

The authors have categorized the identified cybersecurity issues as direct and indirect consequences of COVID-19, as presented in Figure 1.

### Direct Consequences of COVID-19

#### Data Sharing

The most pressing challenges related to contact tracing focus on the balance between sharing and maintaining the privacy of personal data, which is crucial but difficult to achieve [3,4]. Contact tracing apps should not be made available without proper risk assessment and data integrity verification [8]. If data are anonymized, integrity is more difficult to guarantee because anonymized data are more susceptible to undetected interference; thus, these data are not useful and trustable for the proposed health care goal. Data integrity can only be achieved by forfeiting some degree of privacy, ideally to a trusted entity.

The lack of information technology and cybersecurity literacy can also make it more difficult for most individuals to adequately install and use contract tracing apps, while the apps themselves may integrate technical security vulnerabilities and risks [9]. For instance, the most commonly used protocol in tracing apps is Bluetooth, which has known and intrinsic vulnerabilities, such as a lack of boundary control. Bluetooth signals can traverse walls and cars, and individuals who receive the signals may not be actually in contact with an infected person. The balance between frequent false positive or negative results may need adjustment according to the evolution of the pandemic, as it may be safer to obtain more false positives than false negatives, especially if the virus is very contagious [10].

Finally, even if these contact tracing apps gain wide adherence and use, questions arise. What is the effective return or benefit for individual or public health? How can this return or benefit be measured in relation to the loss of privacy? Now is the right time to answer these questions.

#### Fraud and Theft

There has been a great increase in false messages associated with the COVID-19 pandemic [11]. These messages feed on the spread of misinformation, “fake news,” fear, isolation, and lack of awareness to turn the confined population into a vulnerable target for those types of attacks [12] and persuade victims to give away money, personal data, and credentials (eg, phishing, ransomware, false fundraising campaigns) [13]. Further, when people are isolated, they tend to buy more products on the web; therefore, attackers can take advantage of fraudulent product delivery messages. It should be noted that security data breaches performed during this time will not only be exploited now but will have an extremely wide impact in the future, as this exploitation will continue for a long time after the pandemic has subsided.

In drastic times such as the COVID-19 pandemic, other serious issues may arise. International espionage and sabotage can become more common. Recent examples are the lack of proper management of vaccination procedures as well as the reliance on large multinational companies to fairly distribute and sell the vaccines [14,15]. The provision of preventive means and appropriate public policies to detect and avoid these issues is essential to protect people’s lives.

#### Vulnerable Systems

Most health care systems are underbudgeted, use obsolete technology, and are noninteroperable, and they often lack the latest patches and adequate configurations [16,17]. The COVID-19 pandemic has cleared the way for attackers to better exploit these vulnerabilities. The stress placed on these systems is very high; additionally, there is a risk of shortage of equipment due to the high number of hospitalized patients at one time. European Union countries are required to comply with the General Data Protection Regulation (GDPR) to protect personal data. Unfortunately, this is still not common practice, and organizations attempt to address the situation with few or no resources and, most importantly, with no expert knowledge [18].

### Indirect Consequences of COVID-19

#### Managing Other Non–COVID-19–Related Diseases and Emergencies

Although the COVID-19 pandemic is a serious situation worldwide, with the looming threat of collapse of health care systems, it is not possible to put the treatment of other diseases on hold. Patients with chronic, oncological, mental health, obstetrical, and other health care conditions must be treated. Teleconsultation and web-based medical advice are available [19,20], but at what price for patients’ privacy? The security systems in most home infrastructures are not prepared to adequately control and protect personal and sensitive data.

#### Increased Risks to Physical Security and Integrity

One serious consequence of the COVID-19 pandemic is the isolation of a large part of the world’s population. Fifty years ago, when information was only communicated through the mail and landline telephone companies, cybersecurity was not an issue. Currently, however, almost all daily activities have become virtual. Still, in an ideal world where all home infrastructures are secured and people take the necessary precautions to protect their data and physical integrity, cybersecurity will still be an issue. This is due to the complex relationships between humans and technology. Some common examples are listed below:

- Risky behaviors may arise, as simultaneously assessing every interaction and message from different contexts, 24 hours per day 7 days per week, can create stress and lead people to make poor and unsafe decisions.
- Different contexts (eg, personal, professional, familiar, educational) can easily lead to confusion and mistakes.
- Different populations are affected differently (eg, older people, minority groups, children, and adolescents). Words such as cyberbullying, fake profiles, impersonation, trolling and *Zoombombing* (disrupting Zoom conferences) may come to mind [21]. Home infrastructures are not prepared from a security standpoint, and adults working from home are burdened and distracted, leaving younger people more vulnerable.
- People are engaging in frequent telephone or video calls, and they may often forget to consider the environment they are in and who may be listening. From balconies and gardens, or even through doors or walls, information can slip out more frequently than we may think. Espionage and theft can and often do occur undetected [22].
- Unlocked sessions or devices and microphones or cameras connected at unwanted times can share more personal information than they should.

## Required Changes in Cybersecurity

During the pandemic, the world has been experiencing one wave of COVID-19 after another; still, governments, companies, and the public are all focused on returning to their “normal” prepandemic routines. However, in cybersecurity (as in other areas), “normal” involves low budgets, lack of awareness and education, lack of proper infrastructures, and inability to adapt to different uses by various people and in various contexts. “Normal” also means that privacy and security are still among the greatest challenges in human-computer interactions [23].

Change is crucial, and the COVID-19 pandemic has stressed this even more; however, this change is difficult to achieve. Hence, like a small pebble in a large pond, the authors wish to use this viewpoint to disrupt existing ideas and paradigms and promote other perspectives for discussion in cybersecurity as well as its associated technologies and procedures.

Cybersecurity literacy and education are essential, even more so during pandemic times. One way to achieve these goals is generating scientific research, such as this viewpoint, to raise awareness, provide recommendations, and try new or improved solutions. However, the current times demand web-based, easy, fast, accurate, and objective but personalized and meaningful information and education that is adapted to the situation and context and to the target population [24]. Due to the unpredictable nature of human behavior and actions, humans are an important element and the main enablers of the level of cybersecurity that each system can and will have [24].

However, education is not sufficient. People have thrived for thousands of years by successfully using tools, and not because they are experts or have complete knowledge about every tool or activity [25]. Why should their relations with technology be much different? Several factors may come into play in human-device relations (eg, security, usability, design, efficiency, demographics, previous interactions); however, even when these factors are addressed, adequate and secure use of technology may still not be possible. There is a pervasive line that permeates all these relations and factors that is known as *trust*. Although this can be a *feared* (subjective) subject in computer science, trust can be established on the web because technology has a social presence to which people respond [26]. However, research fails to capture the reasons why end users choose to trust or distrust systems [27] and what factors contribute to trust [28]. A solid formalization of computational trust, to explain how relationships develop through interactions across a range of web-based contexts, would provide enhanced web-based security [29]. Researchers and developers should be brave enough to consider trust development within technology design by providing features that support end users in evaluating the trustworthiness of the technology, helping to promote proper use of technology, and minimizing the frequency of security incidents [30].

By addressing the previous issue, much more can be understood in terms of personality traits, tendency to trust, and propensity toward manipulation and victimization in human-device relations. This will certainly enable the implementation of more adequate strategies to address one of the most critical unsolved problems in cybersecurity—social engineering.

Further, advancements in trust in human-device relations can open the way to more confident use of innovative solutions such as high-fidelity digital humans [31]. These advancements can work to promote *second life* or augmented reality contexts and to improve privacy, for instance, of children and adolescents, with their many interactions using videoconferencing tools (eg, homeschooling, exercise, music lessons).

Some of the discussed ideas can take longer to study and implement; however, while this is being done, the authors suggest the use of anonymous “digital twins” to easily and quickly test interactions between users and technology. Mockup interfaces complemented with anonymous surveys available on the web can be quickly developed to test the security, privacy, and usability of a technology by a large sample of people with a wide range of experiences, characteristics, and behaviors.

Limitations of this viewpoint are its space constraints and the fact that it is based on an original paper published in conference proceedings. Because of this, a more technical and detailed discussion about the introduced subjects is not possible.

## Conclusion

This viewpoint has highlighted the many cybersecurity challenges associated with COVID-19; however, none of the identified challenges are new but have clearly been exacerbated by the pandemic. Therefore, the problems existed before the pandemic, and still no adequate solutions are available. Change and disruption needs to occur at the core of human-device interactions and relations, with a focus on trust and on how humans have thrived with each other over thousands of years, even in threatening situations.

We should take this opportunity to face those challenges before they pile on top of the pandemic toll. In extreme situations, it is normal that exceptions need to be made to prioritize specific parts of society or infrastructures. However, this needs to be accomplished in a transparent and controlled way so that after the exceptional situation subsides, people can easily take back their fundamental right to privacy [32], the loss of which has affected so many lives in the past. We must also claim the right of trust in technology, with more appropriate and improved cybersecurity, for a safer and healthier human population.

## Abbreviations

GDPR: General Data Protection Regulation
